# Angiomyolipome hépatique

**DOI:** 10.11604/pamj.2013.16.107.3427

**Published:** 2013-11-19

**Authors:** Karim Ibn Majdoub Hassani, Tarik Souiki

**Affiliations:** 1 Faculté de Médecine et de Pharmacie de Fès, Université Sidi Mohammed Ben Abdellah, Département de Chirurgie, CHU Hassan II, Fès, Maroc

**Keywords:** Angiomyolipome, tumeur bénigne, foie, angiomyolipoma, benign tumor, liver

## Image en medicine

L'angiomyolipome est une tumeur bénigne mésenchymateuse exceptionnelle du foie. Il existe une prédominance féminine (sexe ratio : 2,5/3) avec un âge moyen lors du diagnostic de 50 ans. La localisation hépatique droite est classique. La taille est extrêmement variable allant de 1 cm à plusieurs dizaine de cm. La possibilité de complication hémorragique est réelle notamment pour les tumeurs volumineuses. L'association d'une sclérose tubéreuse de Bournonville, communément admise pour la localisation rénale, est rare pour l'angiomyolipome hépatique (4-6%). Nous rapportons l'observation d'une patiente âgée de 19 ans, qui présente depuis 4 mois des douleurs de l'hypochondre droit à type de pesanteur, évoluant dans un contexte de conservation de l’état général. L'examen clinque objective une énorme masse du l'hypochondre droit débordant au niveau flanc droit. Par ailleurs, il n'existe ni ictère ni d'autres signes associés. Un bilan biologique comportant les enzymes de cytolyse/cholestase, les sérologies hépatitiques et les marqueurs tumoraux (Alpha FP, CA 19-9, ACE) est strictement normal. Un scanner abdominal avait mis en évidence une volumineuse formation tissulaire, de 12 cm au dépend des segments 5 et 6 hépatiques à développement exophytique. Cette formation, bien limitée, comporte également des composantes graisseuses et vasculaires. Le diagnostic d'angiomyolipome est fortement suspecté, sans par ailleurs exclure le diagnostic de CHC sur foie sain ou d'adénome atypique. La résection de la tumeur a été conduite par voie sous costale droite; Les suites opératoires immédiates étaient simples. L'examen anatomopathologique de la pièce opératoire confirme le diagnostic d'angiomyolipome hépatique: prolifération tumorale à triple composante adipeuse, tissulaire et vasculaires, exprimant la HDM 45 et l'actine musculaire lisse. Le suivi en consultation, après trois mois de l'intervention, est sans particularités ; l'examen dermatologique est normal, excluant l’éventualité d'une association à la sclérose tubéreuse de Bournonville. A long terme, aucune surveillance n'est requise compte tenu du caractère strictement bénin de cette tumeur.

**Figure 1:**
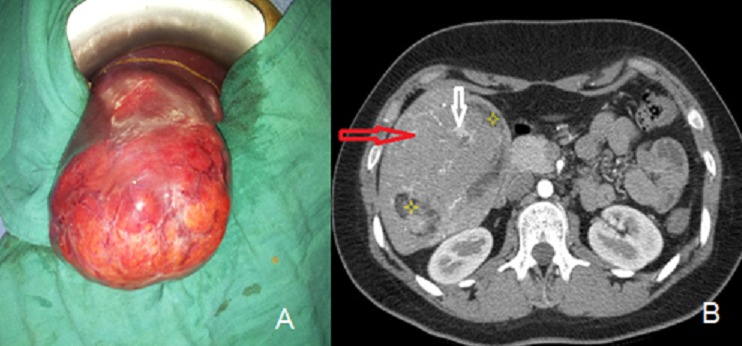
A) Vue opératoire (voie sous costale droite) : Enorme masse tumorale appendue à la face inférieure du foie; B) Coupe scanographique abdominale (L1) : montrant une volumineuse tumeur appendue au foie droit avec triple composante : tissulaire majoritaire (flèche rouge), graisseuse (astérisque) et vasculaire (flèche blanche)

